# Mesenchymal Stem Cells for Treatment of CNS Injury

**DOI:** 10.2174/157015910793358204

**Published:** 2010-12

**Authors:** Michael F Azari, Louisa Mathias, Ezgi Ozturk, David S Cram, Richard L Boyd, Steven Petratos

**Affiliations:** 1 Monash Immunology and Stem Cell Laboratories, School of Biomedical Sciences, Faculty of Medicine, Monash University, Clayton, Vic. Australia; 2 Molecular Neuropathology and Experimental Neurology Laboratory, Health Innovations Research Institute (HIRi) and School of Medical Sciences, RMIT University, Bundoora, Vic. Australia

**Keywords:** Mesenchymal Stem Cell, Spinal Cord Injury, Traumatic Brain Injury.

## Abstract

Brain and spinal cord injuries present significant therapeutic challenges. The treatments available for these conditions are largely ineffective, partly due to limitations in directly targeting the therapeutic agents to sites of pathology within the central nervous system (CNS). The use of stem cells to treat these conditions presents a novel therapeutic strategy. A variety of stem cell treatments have been examined in animal models of CNS trauma. Many of these studies have used stem cells as a cell-replacement strategy. These investigations have also highlighted the significant limitations of this approach. Another potential strategy for stem cell therapy utilises stem cells as a delivery mechanism for therapeutic molecules. This review surveys the literature relevant to the potential of mesenchymal stem cells for delivery of therapeutic agents in CNS trauma in humans.

## INTRODUCTION

The therapeutic efficacy of a large variety of stem and progenitor cells has been assessed in experimental models of central nervous system (CNS) injury and disease. The current status of stem cells in general for the treatment of CNS trauma has recently been reviewed [1-3]. This review focuses on the use of mesenchymal stem cells (MSCs) in particular, with or without genetic modification, for treatment of spinal cord injury (SCI) and traumatic brain injury (TBI).

Earlier transplantation studies employed stem cells in a cell-replacement paradigm. Embryonic stem cells (ESCs) were first used in SCI by McDonald *et al.* in 1999 [4]. In this study they demonstrated appropriate differentiation that was associated with improved functional recovery. Similarly, adult-derived neural stem cells (NSCs) have been used in combination with immunosuppression and growth factors to treat SCI [5]. These investigators showed enhanced survival and differentiation of NSCs into myelinating oligodendrocytes. NSCs exhibited enhanced survival and engraftment resulting in increased numbers of transplant-derived myelinating oligodendrocytes in the spinal cord that correlated with electrophysiological evidence of regeneration through the lesion site. However, stem cells (particularly the more primitive ESCs) may differentiate into inappropriate cell types following transplantation, thereby resulting in tumour formation particularly following allogeneic transplantations [6]. Even the more differentiated NSCs, derived from ESCs, represent heterogenous cell populations and retain the potential for inducing tumours upon transplantation [6, 7]. Transplanted stem cells may also die as a result of the hostile environment of the injured CNS tissue that potentiates endogenous cell death exacerbating the inflammatory response thereby expanding the lesion volume. The viability of neural precursor cells (NPCs) for instance has been reported to be low particularly when grafted into chronic spinal cord lesions necessitating the concomitant administration of growth factors [5]. Furthermore, NSCs can cause inappropriate sprouting and give rise to enhanced pain perception and allodynia following SCI [8, 9]. The degree of allodynia caused by these grafts has also been correlated with the extent of their astrocytic differentiation [8]. However, it has recently been shown that pre-transplantation treatment of astrocytes (derived from glial restricted precursors) with BMP but not CNTF may limit the allodynia that is caused by grafting these cells into the injured CNS [10]. Given the propensity of NSCs to differentiate into glial cells following grafting into the adult injured CNS [11, 12], these cells could be expected to retain the potential to contribute to astrogliosis and the extension of the glial scar. Hence, it has become evident that simple transplantation of stem cells as a cell replacement strategy has limited utility in the treatment of CNS trauma.

## MESENCHYMAL STEM CELLS (MSCS)

In response to the challenges briefly outlined above, alternative transplantation strategies have emerged utilising adult stem cells, particularly MSCs and haematopoietic stem cells (HSCs), that have the intrinsic capacity to track to the site of the lesion within the CNS. Once at the site of the lesion, MSCs can secrete pro-survival factors such as insulin-like growth factor (IGF) and brain derived neurotrophic factor (BDNF), vascular endothelial growth factor (VEGF), granulocyte-macrophage colony stimulating factor (GM-CSF), fibroblast growth factor-2 (FGF2), and transforming growth factor-beta (TGFβ) [13-16]. In addition, these stem cells can be genetically modified to generate peptides or full-length proteins of therapeutic potential such as autocrine factors that promote their own survival [17], as well as the survival or regeneration of neurons [18]. The two major stem cell types that can be isolated from blood and bone marrow are (i) HSCs, and (ii) MSCs also known as mesenchymal stromal cells or fibroblasts. MSCs were first recognised by Alexander Friedenstein and associates in the bone marrow by their ability to form fibroblastic colonies and differentiate into osteoblasts, chondrocytes and adipocytes [19]. MSCs can be easily harvested from a variety of tissues including the umbilical cord blood and adult adipose, bone marrow and skin tissues. They have also recently been isolated from olfactory tissue [20, 21]. In addition MSCs proliferate readily in culture and are amenable to genetic modification. Therefore, they may be ideal candidates for development into appropriate vehicles for delivery of therapeutic molecules. However, it is unclear, at present, whether MSCs isolated from different tissue sources have similar therapeutic potential and which source or isolation protocol is optimal for therapeutic purposes. A recent study for example has suggested that while MSCs from both autologous and allogeneic sources resulted in functional improvement following SCI, autologous MSCs demonstrated greater benefit [22]. In addition, in an experimental SCI paradigm, intravenous administration of adipose tissue-derived MSCs that were differentiated into oligodendrocyte precursor cells demonstrated migration of these cells to the site of the injury, as well as their partial neural differentiation which correlated with behavioural recovery [23]. For a general review of MSCs and their role in health and disease refer to Uccelli *et al*. [24].

### Beneficial Effects of MSC Transplantation

**Fig. (1):**
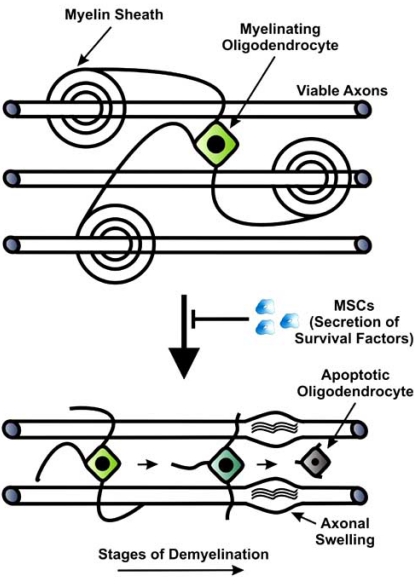
Prevention of oligodendrocyte apoptosis by MSCs. MSC transplantation can reduce apoptosis of oligodendrocytes following CNS injury through secretion of survival factors such as IGF. This results in reduction of demyelination of intact axons as part of the secondary injury mechanisms in which oligodendrocytes retract their processes and then become atrophied and undergo apoptosis. The denuded axons subsequently degenerate and develop axonal swellings in which the tubulin network depolymerises.

Experimental treatments of CNS trauma can be broadly grouped into the two distinct but interrelated strategies of “neuroprotection” and “neurorepair/neuroregeneration”. While neuroprotection refers to inhibition of the death of CNS parenchymal cells following trauma, neurorepair refers to regeneration of severed axons or sprouting of intact axons to innervate denervated targets. MSCs have been used for both of these strategies. Umbilical cord blood is a convenient source of HSCs and MSCs. Interestingly, transplantation of human umbilical cord blood (hUCB) stem cells into the rat spinal cord, 1 week following contusion injury, results in differentiation of these cells into neural cells that display the morphology and immunohistochemical profiles of neurons, oligodendrocytes and astrocytes, along with aiding in the remyelination of denuded axons [25]. However, it is still not clear which molecular factors determine the differentiation profile of MSCs following transplantation. Despite this unresolved issue, it has been reported that transplantation of hUCB cells can downregulate the fas/caspase-3 pathway in both neurons and oligodendrocytes and increase levels of anti-apoptotic proteins, FLICE like inhibitory protein (FLIP) and X-linked inhibitor of apoptosis protein (XIAP) following SCI in rats [26]. Recently, Dasari and colleagues have shown that the anti-apoptotic effects of hUCB cells, at least on cultured rat cortical neurons, are mediated through upregulation of the Akt signalling pathway [27]. Down-regulation of apoptotic and up-regulation of anti-apoptotic molecules (Fig. **1**) has also been reported by this group following transplantation of allogeneic bone marrow-derived MSCs into the contused spinal cord in the rat [28]. These findings are particularly pertinent as we have shown that modulation of anti-apoptotic pathways, by up-regulation of the cellular inhibitor of apoptosis protein-2 (cIAP-2) at the lesion-site, can prevent secondary demyelination and oligodendrocyte apoptosis following SCI in the mouse leading to improvement in locomotor performance [29].

### Homing of Grafted MSCs

MSCs are reported to exhibit a homing capacity, which can be utilised to treat CNS injury. In normal healthy animals they mostly home to the bone marrow [31-33], whereas in animals with active inflammation, MSCs injected intravenously (i.v.) preferentially migrate to the sites of inflammation [34, 35]. Acute single-dose intravenous injection of MSCs however, may not be an effective mechanism to deliver these cells to the injured CNS, as it has recently been shown in the rat following unilateral cortical impact TBI, that over 96% of the injected cells become trapped in the lungs and do not reach the arterial circulation [36]. Intravenously injected human MSCs have been reported to localise to peri-lesional parenchyma following acute weight-drop TBI in rats, particularly when two i.v. injections are performed instead of one [37, 38]. MSCs may therefore be used for tissue directed immunosuppression, or delivery of pro-survival or regenerative molecules to the lesion site, following CNS injury.

### Neural Differentiation of Grafted MSCs

It has been reported that when bone marrow-derived MSCs are cultured under specific conditions such as in the presence of EGF or BDNF, they develop neuronal morphologies and begin to express neural markers such as Nestin, GFAP and NeuN [39]. Other investigators have used 2% dimethylsulfoxide (DMSO) to induce MSCs to express neuronal markers such as NeuN, NSE and tau [40]. These findings have been interpreted as the ability of MSCs to differentiate into neurons, hence suggesting a potential for their use in cell replacement following CNS injury and disease. On the other hand, more recent evidence suggests that morphological differentiation of MSCs into neurons and their expression of neural proteins in culture, may represent artefacts of specific culture conditions such as serum withdrawal that cause F-Actin retraction and cytoplasmic collapse rather than neurite extension [41]. Nevertheless, Tondreau and colleagues have recently found significant upregulation of neural genes and downregulation of chondrogenic, osteogenic, adipogenic and myogenic genes in neurally differentiated MSCs as demonstrated by microarray analysis [42].

**Fig. (2):**
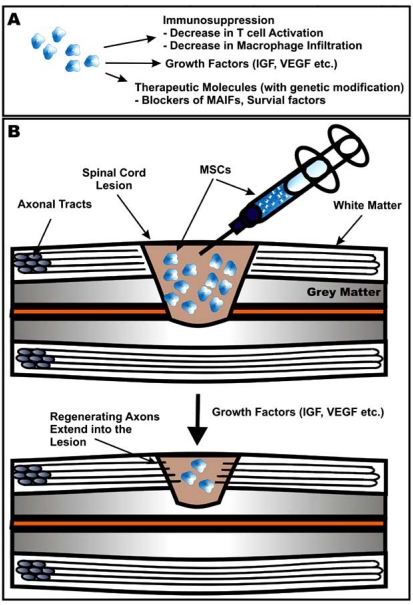
Therapeutic potential of MSCs in CNS trauma. **(A)** MSCs have several properties that can be harnessed for regenerative medical approaches such as their ability to suppress the immune system and secrete growth factors, and their capacity for being used as cellular vectors for therapeutic molecules such as peptides that inhibit myelin derived inhibitory factors (MAIFs) that prevent axonal regeneration. **(B)** Transplantation of MSCs, particularly ones that are genetically manipulated to secrete neurotrophic factors such as BDNF, can result in regeneration of injured axons and their extension into the lesion site, and reduction of the size of the spinal cord lesion.

Even though expression of the dopaminergic neuronal marker, tyrosine hydroxylase (TH), has been reported in scattered MSCs engrafted into the striatum in the MPTP model of Parkinson’s disease [43], other investigators have found no evidence of neural differentiation, in spinal cord or brain injury [44]. Vallieres and colleagues, for instance, injected GFP-expressing bone marrow cells in adult irradiated rats and investigated their cellular phenotypes 1-12 months subsequently and found that even following brain injury these cells remained haematopoietic and did not differentiate into neural cells [45]. Although MSCs can be found months after administration they are only found in small numbers, despite continued enhancement of neurological function at these time-points [46]. In addition, many of these MSCs lodge in non-CNS tissues. This suggests that improved neurological outcomes may not be due to engraftment of MSCs at the lesion site and their differentiation into neural cells, but rather to secretion of soluble factors by MSCs that may limit cell death in the CNS or promote endogenous progenitor cell proliferation [47]. This notion is supported by the finding that when conditioned medium from adipose MSCs is administered intravenously following hypoxic-ischaemic brain injury in neonatal rats, hippocampal and cortical cell death is greatly reduced enhancing locomotor recovery [14]. Some of the soluble factors suggested to be responsible for this are IGF, VEGF, nerve growth factor (NGF), and hepatocyte growth factor (HGF) [48] (Fig. **2A**). In summary, the issue of neural and/or neuronal differentiation of MSCs remains controversial and has been reviewed in more detail elsewhere [49].

### Pre-transplantation Genetic Engineering of MSCs 

A viable strategy to further enhance soluble factor production by MSCs is genetic modification. In a recent report, MSCs that overexpressed the HSC growth factors, GM-CSF and stem cell factor (SCF), were shown to promote the engraftment of co-transplanted cord blood HSCs in non-obese diabetic/severe combined immunodeficiency (NOD/SCID) mice [50]. MSCs have been genetically modified to produce a wide variety of neurotrophic factors including neurotrophin-3 (NT-3), BDNF, and NGF, followed by transplantation into the site of spinal cord lesion. MSCs, genetically modified to overexpress NT-3, can promote axonal growth in the corticospinal tract following SCI [51]. Robust promotion of rubrospinal, vestibulospinal, and reticulospinal tract axonal regeneration, as shown by neuronal tracing methods, is also possible in the chronically injured spinal cord when BDNF is delivered by MSCs four weeks following hemisection injury [52]. When BDNF-producing MSCs are transplanted into the site of rubrospinal tract ablation [18] or lateral funiculus lesions [53] in the adult rat spinal cord, they not only reduce neuronal loss in the red nucleus, but also promote axonal regrowth and lead to locomotor recovery (Fig. **2B**).

Furthermore, when human MSCs that are modified to express BDNF by transfection with an adenovirus vector [54, 55], are transplanted into rats with transient middle cerebral artery occlusion (MCAO), they can cause improved recovery from ischaemia after 7-14 days, and a reduction in apoptotic cell numbers in the ischaemic penumbra. Moreover, MSCs that overexpress BDNF and NT-3 together, not only promote recovery of hindlimb movement but also bladder function following contusion injury of the thoracic spinal cord in rats [56]. However, the pro-survival effects of transfected MSCs may be population specific depending on the neurotrophin they express. This notion was highlighted when MSCs expressing either NT-3 or NGF were transplanted into the injured spinal cord of adult rats, and the cells expressing NT-3 were able to prevent neuronal loss in Clarke’s nucleus whereas NGF expressing MSCs exhibited partial protection of these neurons [57]. There is evidence that the cell specificity of neurotrophins also applies to sprouting of uninjured neurons in the spinal cord, as sensory neurites can sprout in response to MSC-delivered NGF, but not when FGF2 is delivered by these cells [58]. The differential responses of disparate neuronal populations to MSC-delivered neurotrophins (using constructs for overexpression) were also reported by Nakahara and colleagues who found that motor neurites did not sprout in response to NGF, NT-3 or FGF2 in the absence of injury while sensory neurites did, and that injury was also required for sensory neurites to sprout in response to BDNF [59]. Furthermore, severed axons located in the long descending spinal tracts seem to require specific neurotrophins in a differential manner. Whereas rubrospinal tract fibres regenerate in response to administration of BDNF, due to their expression of the TrkB receptor [60, 61], corticospinal tract fibres require a combination of BDNF, NT-3 and GDNF, together with peripheral nerve implantation for optimal regrowth [62]. Therefore, the requirements of the injured CNS tissue for neurotrophic factors are complex and cell type-specific. This mandates greater sophistication in terms of the design of therapeutic strategies using precise combinations of neurotrophic factors and specific modes of delivery to promote targeted neuronal regeneration.

The secretion of soluble pro-survival molecules by MSCs can be further enhanced by the trophic factors at the injury site as MSCs cultured with supernatants from ischaemic brain extracts upregulated the production of these factors [48]. On the other hand, factors within the injured spinal cord may down-regulate the expression of artificially introduced BDNF in modified MSCs, as this expression has been reported to decrease within two weeks following injury, while it could be resumed following harvest and re-culture [63]. Therefore, the complex interaction between MSCs and the tissue milieu of the injured CNS remains to be fully explored.

### Immunomodulatory Effects of MSC Transplantation

There is evidence to suggest that MSCs are immunosuppressive and that they are non-immunogenic stem cells that may cross HLA barriers. These cells do not initiate an allogeneic response *in vitro* and are not rapidly rejected *in vivo* [64]. This may reduce the necessity to use concomitant immunosuppressive treatment, as the immune system may not recognise the low HLA-class 1 expression on the MSCs. In addition, these cells secrete immunomodulatory molecules that arrest T cells in the G0 phase of the cell cycle that may also aid MSCs to escape immune recognition. MSCs can inhibit the pro-inflammatory cytokine interleukin-1 (IL-1), by secretion of IL-1 receptor antagonist (IL-1RN), and also inhibit production of another major pro-inflammatory cytokine, tumour necrosis factor-alpha (TNF α), by activated macrophages [65].

**Fig. (3):**
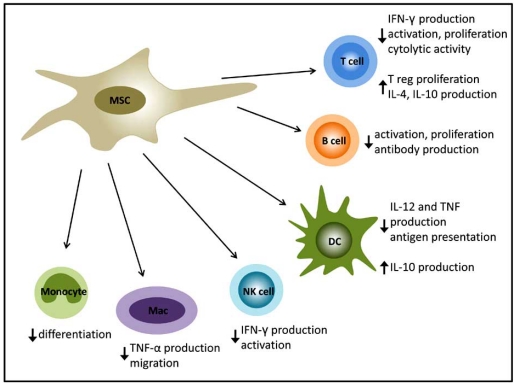
MSCs have the capacity to modulate the immune response by reducing pro-inflammatory immune cell function and the production of inflammatory cytokines whilst promoting regulatory T cell proliferation and secretion of IL-4 and IL-10.

MSCs have been demonstrated to have the capacity to induce a variety of specific responses from the different immune cell types (Fig. **3**). In fact the anti-inflammatory properties of MSCs are sufficiently potent to have been utilised to treat Graft-versus-Host Disease (GvHD) in patients and there is also evidence to suggest that they support haematopoiesis, enhancing HSC transplantation [68] and immune recovery *in vivo* [69]. Comprehensive reviews of the literature on this subject are available [24, 66, 67]. Hilmes *et al.* have shown locomotor recovery and moderate regeneration through the lesion site, following moderate weight drop contusion SCI in the rat, after transplantation of bone marrow derived stromal cells. However, at 11 weeks after injury, very few of these cells survived in the spinal cord [46]. This finding may highlight the role of the host immune system in rejecting transplanted MSCs in the absence of immunosuppression. To circumvent this challenge, Tobias and colleagues employed an innovative strategy in which they encapsulated MSCs in Alginate-poly-L-ornithine to protect them from the host immune system and were able to obviate the need for immunosuppression [63].

### Safety of MSC Transplantation

Human safety of MSC transplantation has been shown in stroke patients who also exhibited improved neurological function [70]. MSC transplantation also results in moderate improvement in nerve conduction velocities in patients with Metachromatic Leukodystrophy and Hurler syndrome [71]. The first clinical trial of MSCs in humans with SCI showed improved neurological function following autologous whole bone marrow transplantion administered to the site of injury in combination with intravenous GM-CSF [72]. Moreover, in a clinical trial using autologous MSCs injected into the vertebral artery the Sykova group have shown clinical improvement as measured by ASIA score in patients with subacute SCI [73]. In addition, in a recent clinical trial, combined intraparenchymal and intravenous administration of MSCs resulted in promotion of neurological recovery in TBI patients [74].

### Potential Caveats of MSC Transplantation

MSCs, by definition, have the potential to differentiate into osteoblasts, adipocytes and chondrocytes. Osteoblastic differentiation of intravenously injected MSCs has recently been reported in lung tumours in mice [75]. Hence, while there are no reports of non-neural differentiation of MSCs following transplantation into the injured CNS, this possibility cannot be ruled out. Being of connective tissue origin, a possible caveat of MSC transplantation following CNS trauma is that these cells could potentially contribute to the formation of the glial scar. While this possibility cannot be ruled out, Veeravalli and colleagues have recently shown that hUCB transplantation is associated with a reduction of the glial scar and an up-regulation of matrix matelloproteinase-2 (MMP-2), a gelatinase that has the capacity to degrade scar tissue [30]. However, it is not clear to what extent the MSC component of hUCB is responsible for these pro-regenerative effects.

Another potential caveat of therapies that are designed to induce sprouting and axonal regeneration, including transplantation of MSCs that secrete growth factors, is that they may cause inappropriate sprouting that causes or enhances neuropathic pain [76]. At present, it is not possible to therapeutically induce axonal regeneration in motor axons following CNS injury while sparing sensory axons. This is due to similarities in molecular mechanisms that govern axonal growth in both sensory and motor neurons.

MSC transplantation particularly following genetic modification is a promising strategy in treating CNS trauma. MSCs reside in a range of tissues that are easily accessible such as adipose tissue, skin, bone marrow, and even peripheral blood. They can be therapeutically used in an autologous manner obviating the need for concomitant immunosuppression. MSCs proliferate readily in culture and are amenable to genetic modification. Unlike ESC-derived cells, MSCs represent an adult cell population that do not give rise to tumours upon transplantation. Furthermore, MSCs track to the site of the lesion in the injured CNS where they secrete pro-survival factors. The combination of these properties of MSCs may make them ideal candidates for development into cell therapy strategies for neurotrauma in humans, particularly as delivery vehicles for therapeutic molecules.
